# Hemodiafiltration for children with stage 5 chronic kidney disease: technical aspects and outcomes

**DOI:** 10.1007/s00467-024-06285-w

**Published:** 2024-02-13

**Authors:** Charlotte Ahlmann, Lynsey Stronach, Kathryn Waters, Kate Walker, Jun Oh, Claus Peter Schmitt, Bruno Ranchin, Rukshana Shroff

**Affiliations:** 1 University College London Great Ormond Street Hospital and Institute of Child Health, London, WC1N 3JH UK; 2 University Medical Center Hamburg-Eppendorf, Hamburg, Germany; 3 Centre for Pediatric and Adolescent Medicine, University of Heidelberg, Heidelberg, Germany; 4 Hôpital Femme Mère Enfant, Hospices Civils de Lyon, Université de Lyon, Lyon, France

**Keywords:** Hemodialysis (HD), Hemodiafiltration (HDF), Children, Blood pressure, Cardiovascular disease, Growth

## Abstract

Despite significant medical and technical improvements in the field of dialysis, the morbidity and mortality among patients with chronic kidney disease (CKD) stage 5 on dialysis remains extremely high. Hemodiafiltration (HDF), a dialysis method that combines the two main principles of hemodialysis (HD) and hemofiltration—diffusion and convection—has had a positive impact on survival when delivered with a high convective dose. Improved outcomes with HDF have been attributed to the following factors: HDF removes middle molecular weight uremic toxins including inflammatory cytokines, increases hemodynamic stability, and reduces inflammation and oxidative stress compared to conventional HD. Two randomized trials in adults have shown improved survival with HDF compared to high-flux HD. A large prospective cohort study in children has shown that HDF attenuated the progression of cardiovascular disease, improved bone turnover and growth, reduced inflammation, and improved blood pressure control compared to conventional HD. Importantly, children on HDF reported fewer headaches, dizziness, and cramps; had increased physical activity; and improved school attendance compared to those on HD. In this educational review, we discuss the technical aspects of HDF and results from pediatric studies, comparing outcomes on HDF vs. conventional HD. Convective volume, the cornerstone of treatment with HDF and a key determinant of outcomes in adult randomized trials, is discussed in detail, including the practical aspects of achieving an optimal convective volume.

## Introduction

Children on dialysis have a high mortality [1], a significant burden of comorbidities, and report a poor health-related quality of life compared to their peers [2]. Patients on long-term hemodialysis (HD) often have volume overload and hypertension, anemia, mineral dysregulation, endothelial dysfunction, inflammation, and oxidative stress, that together worsen the cardiovascular risk profile [3, 4], and lead to hypertension, bone disease, and growth failure. Randomized trials in adults suggest that increasing the dose of conventional HD treatment or using high-flux dialysis does not improve outcomes. In contrast, hemodiafiltration (HDF), a dialysis modality that combines the principles of diffusion and convection, enhances the clearance of middle molecular weight uremic toxins, potentially contributing to improved outcomes [5]. Greater hemodynamic stability contributes to fewer dialysis-related symptoms and better tolerance of dialysis [6]. Two trials in adults have reported improved survival in patients treated by HDF compared to high-flux HD [7, 8].

HDF was introduced in pediatric practice in the 1970s by Fischbach et al. who showed that growth retardation in children with stage 5 CKD could be reversed by daily HDF [9]; however, it was unclear if daily dialysis or HDF therapy per se resulted in the improved growth. More recently, a large prospective study across Europe and North America, the HDF, Hearts, and Height (3H) study, that compared conventional HD with HDF, both delivered three times a week, showed that children on HDF have an attenuated cardiovascular risk profile and improved growth [10]. Also, an improvement in quality of life is shown in several independent studies reporting a shorter post-dialysis recovery time, less fatigue, and improved life participation with HDF compared to HD treatment [9–11].

This educational review examines the current state of knowledge regarding HDF in pediatric patients. Technical aspects of performing HDF in children and the impact of HDF on important clinical outcomes, such as cardiovascular disease, growth, bone health, inflammation, and quality of life are discussed.

## Types of extracorporeal dialysis

**Fig. 1:**
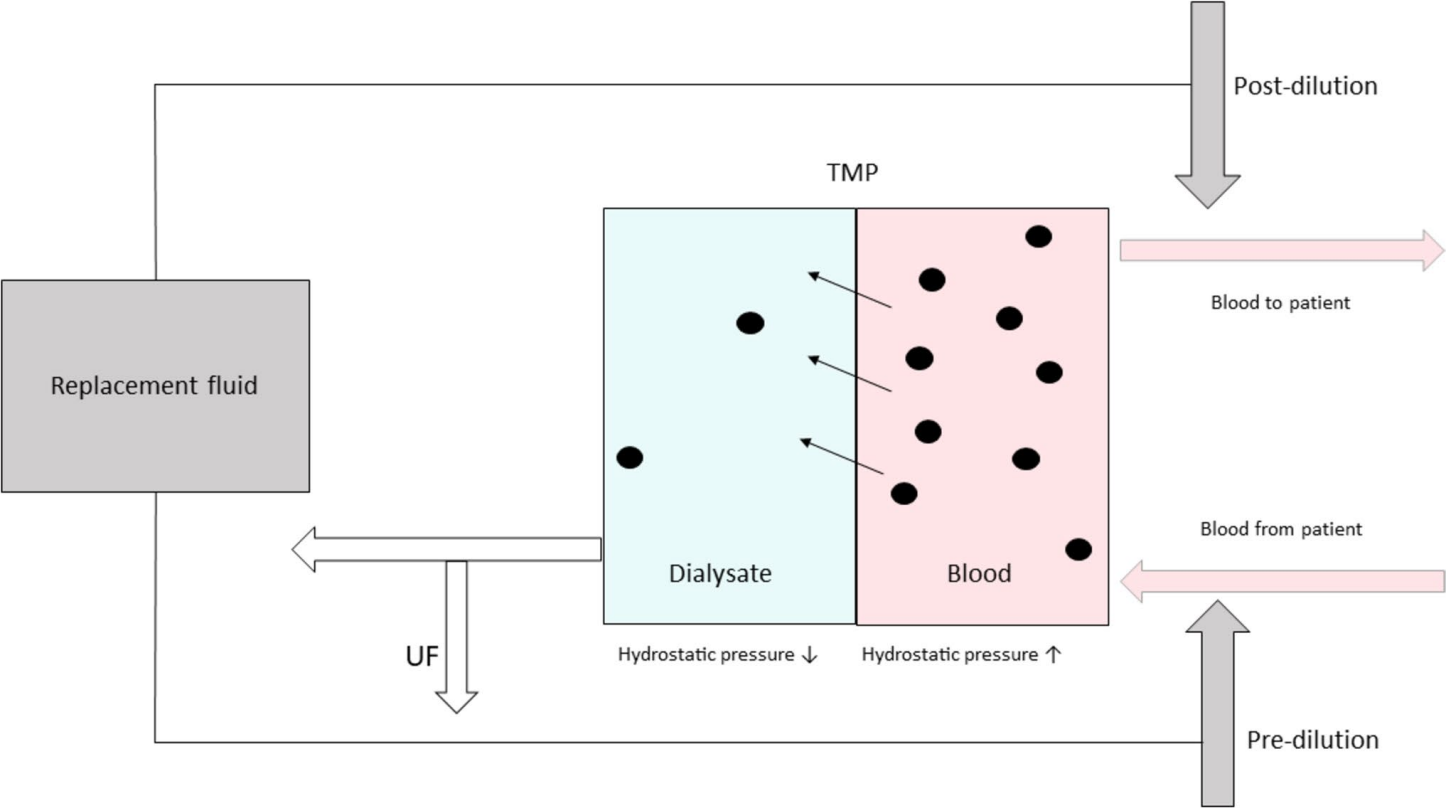
Technical aspects of hemodiafiltration. *UF*, ultrafiltration; *TMP*, transmembrane pressure

Extracorporeal dialysis includes three main techniques: HD, hemofiltration (HF), and HDF (Fig. 1):

I. *Conventional HD* works by the physical principle of diffusion, meaning solutes are removed along a concentration gradient across a semipermeable membrane [5, 12, 13]. Low molecular weight solutes, such as urea, creatinine, and potassium, that have a molecular weight of less than 500 Daltons [14], are eliminated most efficiently by diffusion [12, 13]. The surface area and sieving coefficient of the dialyzer and blood and dialysis fluid flow determine the quality of HD provided. Sieving coefficient is a key determinant of the mass transfer area coefficient (K_0_A) and consequently the solute permeability of a dialysis membrane.
II. *HF* is mainly used for rapid fluid removal in intensive care units. It allows for a small and variable amount of convective transport of uremic toxins depending on the prescription, but at the infusion rates and session lengths used in chronic maintenance therapy, HF delivers poor clearance of small molecules like urea [15], and should not be used for long-term dialysis. It is not discussed further in this review.
III. *HDF* combines the diffusive solute removal of HD with the convective clearance provided by HF [5, 12, 13, 16]. The convective transport occurs when a fluid stream is driven across a semipermeable membrane by a transmembrane pressure (TMP) gradient, carrying the solutes along with it, also called solute drag [5, 16]. The UF coefficient (K_UF_) is used to describe the effectiveness of a membrane to ultrafiltrate fluid. K_UF_ is Q_UF_/∆P (volume of UF per unit time, divided by the transmembrane pressure [TMP]).

**Fig. 2:**
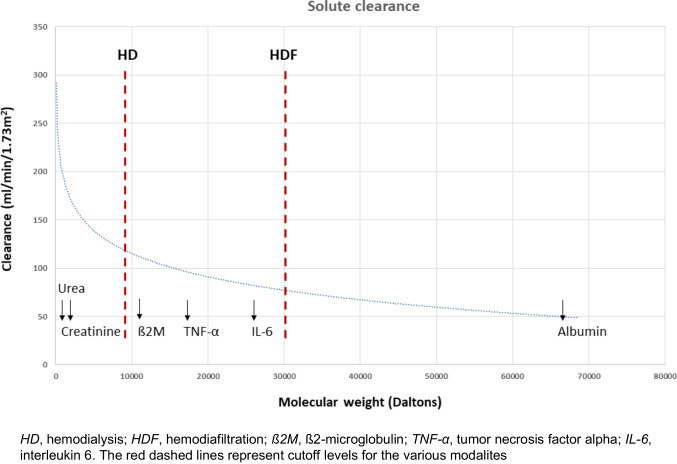
Relationship between molecular weight and clearance

Convection removes a wide range of middle molecular weight uremic toxins up to 50,000 Daltons (for comparison, albumin has a molecular weight of 66,500 Daltons). ß2-microglobulin (ß2M), the prototype middle molecule (molecular weight 11,800 Daltons), is used as a surrogate for middle molecular weight uremic toxin clearance in HDF (Fig. 2).

## Definition of HDF therapy

The European Dialysis Working Group (EUDIAL) of the European Renal Association defines HDF as a blood purification treatment that provides both diffusive and convective solute removal by ultrafiltration of 20% or more of the blood volume processed through a high-flux dialyzer, with sterile replacement fluid infused directly into the patient’s blood to maintain fluid balance [17]. By the online filtration of standard dialysis fluid though a series of bacteria- and endotoxin-retaining filters, sterile replacement fluid is obtained in large volumes; this is called online HDF [5]. By definition, a dialysis membrane is classified as high flux if it has an ultrafiltration coefficient greater than 20 mL/h/mmHg TMP and a sieving coefficient for β_2_M of greater than 0.6. The convective component of HDF therapy allows for a greater removal of middle and large molecular weight uremic toxins than that achieved with low or high flux HD [5, 17]. Of note, there is no clinically significant removal of protein-bound uremic solutes (such as p-cresyl sulphate and indoxyl sulfate) by HDF or HD [18].

## Modalities of HDF

During HDF, a large volume of ultrapure water is infused into the patient to achieve convective clearance [13, 16, 19]. There are two main modalities of HDF depending on the point of infusion of the replacement fluid relative to the dialyzer (Fig. 1):

I. *Pre-dilution HDF*: When the replacement fluid is infused upstream of the dialyzer, the modality is called pre-dilution HDF. Pre-dilution HDF requires the infusion of substitution fluid at 100% of the blood flow rate. Of note, the dilution of the blood with the replacement fluid will reduce the clearance by diffusion and convection because it reduces solute concentrations in the blood compartment [13]. To achieve equivalent clearances as post-dilution HDF, the convective volume with pre-dilution HDF needs to be 2 to 3 times greater in pre-dilution as compared to post-dilution HDF [5]. Pre-dilution HDF reduces hemoconcentration and reduces the risk of clotting in the filter [13].
II. *Post-dilution HDF*: When the replacement fluid is infused downstream of the dialyzer, it is called post-dilution HDF. Post-dilution HDF is the most commonly used HDF technique in adults and children [5]. However, at high ultrafiltration rates, post-dilution HDF can lead to hemoconcentration and the deposition of plasma proteins on the dialyzer membrane, that in turn can occlude blood channels within the dialyzer and raise the TMP. This can reduce clearance and also increase the risk of clotting of the extracorporeal circuit [5]. The degree of hemoconcentration that occurs depends on the filtration fraction (see section below). A filtration fraction (FF) up to 30–35% of the blood flow rate is recommended to prevent hemoconcentration, that in turn can lead to circuit loss, as well as too much protein deposition on the dialyzer membrane. An optimal FF can be achieved with systems that detect and automatically adjust the FF based on TMP and adapt to ultrafiltration flow rate measurements [17].

***Filtration fraction*** (FF) is a term unique to convective therapies and determines the convective volume [17]. FF is defined as the ratio of the ultrafiltration (UF) rate to the plasma water flow rate [17]. Blood flow rate (*Q*_*b*_) which is indicated on all dialysis machines is often used as a surrogate for plasma water flow rate. Thus, FF may be expressed as the ratio of the total UF to the total blood flow (*Q*_*b*_) [or the plasma flow (*Q*_*p*_)] that is delivered to the filter.

$$FF=\frac{{\text{QUF}}\left({\text{Total}}\right)}{Qp}$$

where

UF: total amount of plasma water removed from the patient
*Q*_*p*_: *Q*_*b*_ (1–hematocrit)

In clinical practice, net UF is the sum of the desired intradialytic weight loss in kilograms and the amount of fluids administered during treatment. The higher the FF, the greater the convective volume extracted from the blood. A safe and effective filtration fraction is up to 30–35% of the blood flow rate, as the risk of hemoconcentration within the filter increases proportionately to an increase in FF.

## Technical aspects of hemodiafiltration

Essential requirements for performing HDF include (1) dialysis machines with accurate ultrafiltration control; (2) high-flux dialyzers; and (3) “ultrapure” water for replacement of convective volume.

I. *Dialysis machines with accurate UF control*: HDF requires a suitable machine and software for pediatric use, essentially having the capability of producing ultrapure water and offering very accurate ultrafiltration control. Safety in terms of monitoring and accurately controlling the fluid balance during dialysis is of particular importance in children, as fluctuations in this can lead to potentially life-threatening hypo- or hypervolemia [20]. Dialysis machines are often not suitable or not approved for use in small children, with currently not a single HD or HDF machine available for children weighing less than 10 kg, even though they make up 2–9% of children requiring dialysis [21]. Currently available machines in Europe which are suitable for HDF in children are manufactured by Gambro and Fresenius Medical Care. Unfortunately, the manufacture of both Fresenius 5008 machines as well as the Gambro AK200 Ultra-S has been discontinued recently, so that only the Fresenius 6008 machine is available for HDF in children with 10 kg body weight or higher using a pediatric circuit [20, 21]. Other dialysis machines that can be used for HDF in children around the world include the Fresenius 4008 machine (> 15 kg), the Nikkiso-DBB-EXA and 200Si (> 20 kg), the Baxter Artis Physio (> 25 kg), and the Braun dialog iQ and Nipro surdialX (30 kg) [21].

Important aspects of the HDF machine include:

1. Volume control: Precise management of ultrafiltration and replacement fluid rates is crucial to prevent hypovolemia or fluid overload. Modern HDF machines have advanced ultrafiltration control systems to ensure precise fluid removal and infusion rates. This precision is vital, given the larger volumes processed in HDF compared to HD. Also, optimized flow rates are necessary for efficient solute removal. Advanced machines offer automated adjustments of removal flow rate based on real-time data of hematocrit and or hemoconcentration.
2. Temperature control: systems that monitor and regulate blood temperature can prevent hypothermia. This is especially important given the large volume of replacement fluid infused into the patient.
3. Pressure monitors: continuous monitoring of the TMP and venous pressures can prevent filter clotting and enhance solute removal efficiency. A continuous optimization of replacement fluid flow rate can be regulated in response to variations in the actual blood flow rate, hemoconcentration, and performance of the membrane [22]; this is achieved by the AutoSub mode on Fresenius devices, the UltraControl on Baxter machines, or the TMP-SUB control on Nikkiso devices.

II. *High-flux dialyzers*: Important characteristics that are used to describe a dialysis membrane include its ultrafiltration coefficient (K_UF_) and mass transfer coefficient (K_0_A; both described above) as well as the sieving coefficient, retention onset, cutoff, and adsorptive capacity. Details of dialysis membranes are beyond the scope of this review, but readers are referred to an excellent recent publication on the subject []. Of note, the newer medium cutoff dialyzers (that enhance large middle molecule clearance up to a molecular weight of 45,000 Daltons) should not be used for HDF as they can lead to a very high albumin loss. It is important for practitioners to check dialyzer specifications to ensure they are appropriate to use for HDF treatment.

Highly permeable membranes, characterized by a coefficient of ultrafiltration (K_UF_) > 20 mL/h/mmHg TMP and a sieving coefficient (S) of > 0.6 for ß2M, are required for the clearance of middle molecular weight uremic toxins by HDF. The optimal dialysis membrane for HDF must have a K_UF_ > 50 mL/h/mmHg TMP, a sieving coefficient for β_2_M of greater than 0.6, biocompatibility and endotoxin-retaining capacities [17]. Biocompatible membranes, such as synthetic polysulfone or polyacrylonitrile, are preferred due to their lower activation of the complement system. As with HD therapy, the surface area of the dialyzer should be at least equal to the patient’s body surface area, but not more than 120% of the patient’s body surface area as the dialyzer surface area correlates with its volume, and a large extracorporeal volume will exsanguinate the patient and increase the risk of intradialytic hypotension.

III. *Ultrapure water*: An essential prerequisite for the performance of HDF is the availability of sterile and pyrogen-free replacement fluid, also called “ultrapure” water, that is infused directly into the patient’s bloodstream [17, 20]. “Ultrapure” water is essentially of the same standard of sterility as intravenous fluids, and by definition must contain less than < 0.1 colony-forming unit (CFU)/mL of bacteria, and have endotoxin levels lower than < 0.03 endotoxin units (EU)/mL [13, 16, 23]. The infusate used to be provided in pre-packaged bags, but is now produced directly by the dialysis machine by filtering the dialysis fluid through bacteria- and endotoxin-retentive filters, thereby allowing far larger volumes of replacement fluid; this is known as online hemodiafiltration (OL-HDF) [13, 16]. The International Organization for Standardization (ISO) has published a series of standards addressing fluids for extracorporeal therapies. Specifically, ISO 11663:2009, *Quality of dialysis fluid for hemodialysis and related therapies*, requires that replacement fluid used for HDF be sterile and pyrogen-free as defined above [24]. Online hemodiafiltration is not available for adults or children in some countries including the USA partly because regulatory authorities remain concerned about the safety and sterility of the large volumes of substitution fluid infused directly into the bloodstream to maintain fluid balance.

Importantly, although these quality standards are set for HDF only, a process called back filtration (whereby dialysis fluid enters the blood compartment due to a pressure gradient within the dialyzer) can occur in high-flux dialyzers, leading to chronic low-grade endotoxemia, a known cardiovascular risk. Hence, it would be ideal to use ultrapure water for HD treatment that is performed with high-flux dialyzers, too.

Microbiologic analyses to check water quality are recommended at least 3 × monthly.

Other aspects of HDF therapy to consider are:

I. *Dialysate composition* is similar for HD and HDF treatments, but careful attention to dialysate sodium concentration is important for hemodynamic tolerance and to maintain sodium balance. The dialysate sodium concentration required for HDF is lower than in conventional HD, to avoid the risk of sodium loading from the high volume of substitution fluid used. This is particularly important when high convective volumes are infused, as with pre-dilution HDF. A high dialysate sodium is likely to cause fluid overload with hypertension and increased thirst, although it may improve hemodynamic tolerance. In contrast, a low dialysate sodium enables additional sodium removal by diffusion, but it may be associated with a risk of intradialytic hypotension and disequilibrium syndrome. Also, sodium profiling is not recommended in HDF as there is a risk of sodium loading. In the authors’ experience, keeping the dialysate sodium level within 5 mMol less than the blood sodium level in post-dilution HDF allows for optimal fluid removal without compromising hemodynamic stability.
II. *Substitution modes*: Some HDF machines such as the Fresenius 5008 and 6008 machines have automatic substitution modes called AutoSub™ and AutoSub Plus™. In AutoSub Plus, the substitution rate is automatically regulated in response to variations in patient- and treatment-related parameters throughout the dialysis session, thereby optimizing the convective clearance. While it is possible to manually override this, there is no advantage to it.
III. *Temperature control*: Dialysis machines have heating coils and integrated temperature sensors (for the dialysate and patient’s body temperature) to control thermal exchanges during the dialysis session and perform isothermic dialysis. However, the extracorporeal circuit can cool rapidly, particularly when large substitution volumes and dialysate flow rates are used as with HDF, and this results in an innate cooling during HDF. Cooling prevents vasodilatation and intradialytic hypotension, thereby improving the tolerance to dialysis. The dialysate temperature is set at or within 0.5 degrees of the patient’s body temperature for HDF therapy, and depends on the child’s tolerance to cooling.

## The importance of optimizing convective volume

A high convective volume is the cornerstone of effective HDF treatment. The convective volume is the sum of the net ultrafiltration volume (i.e., the amount of fluid removed during a dialysis session based on the inter-dialytic weight gain) and the amount of sterile replacement fluid infused into the patient (also called substitution volume). A ß2M reduction ratio over 80% demonstrates the efficiency of middle molecular weight uremic toxin clearance [25], and thus implies that an optimal convective volume is applied. Improved survival in patients on HDF is demonstrated only when the convective volume exceeds 20 L/session; this has been shown through randomized controlled trials (RCTs) in adults [7, 8, 26–28] and a pooled individual participant data analysis [29]. Based on these RCT data, any treatment that processes less than 20% of total blood volume does not qualify as HDF [17].

In children, a target convective volume of 13–15 L/m^2^/session in post-dilution mode is aimed for, derived from adult studies [30]. If the patient has a high hematocrit, pre-dilution HDF may be considered [31]. Clinicians should strive for an optimal blood flow, dialyzer surface area, and dialysis time to achieve the highest possible convective volume. An optimal convective volume is the convective volume that is shown to improve survival in adult RCTs normalized to body surface area for children. This can be safely achieved with automated control of substitution flow rate with maximizing filtration fraction throughout the session, and it is easier to manage for the dialysis nurses.

## How to optimize the convective volume—a practical guide

Selecting the “HDF mode” on a dialysis machine will not automatically result in high convective volumes. To optimize the convective volume, it is important to understand its determining factors. It has been shown that rather than patient characteristics such as serum albumin, hematocrit, or body size, the blood flow rate as well as the treatment time play a greater role in determining convective volume [27, 32]. Also, the recently published CONVINCE trial [8] has shown that patients could consistently achieve very high convective volumes of > 25 L/session, although it was a pre-selected group that was recruited to the trial [27].

Optimizing the convective volume requires:

1. A high blood flow rate (as the filtration fraction is strongly influenced by the blood flow).
2. Setting an FF of up to 30–35% of the blood flow rate.
3. Optimization of substitution volume which can be performed manually or by automated programs in new dialysis machines. Practical tips to optimize convective volume are shown in Table 1. Of note, both the substitution volume and the FF can be set on machines designed to perform HDF.
4. Monitoring of substitution volume obtained in each session.

**Table 1:** How to optimize the convective volume in HDF

	Practical Tips	Description
(i)	Optimal vascular access	Both central venous catheters and fistulas can be used for HDF, but higher blood flow is usually achieved through a fistula
(ii)	Needle size	The choice of a fistula needle is based on type, vintage, expansion of the access, bleeding susceptibility, and patient preference Use the largest needle size suitable for the access type, with exceptions for initial cannulation
(iii)	Avoid single-needle HDF	Single-needle HDF should not be performed. In these systems, arterial and venous line clamps are alternately opened and closed, leading to a mean blood flow lower than with double-needle procedures. This may also cause variable transmembrane pressure and FF fluctuation, leading to inadequate and unpredictable convective volumes
(iv)	Access recirculation	An increase in blood flow rate can lead to recirculation, especially in cases of insufficient arterial inflow or obstruction in the venous outflow tract. Increasing the convective volume through recirculation is inefficient and undesirable
(v)	Effective vs. set blood flow rates	The true blood flow rate might be lower than the set value. The discrepancy grows with higher blood pump speeds due to partial tube collapse at more negative pre-pump pressure. The type of access also plays a role: for instance, a set blood flow resulted in lower actual flow in a CVC compared to an AVF
(vi)	Anticoagulation	Adequate anticoagulation with either unfractionated heparin or low molecular weight heparin is essential due to the risk of hemoconcentration and clotting within the dialyzer. The optimal dosing is not well-defined, but higher doses than typically used with both low-flux and high-flux HD might be required due to the likelihood of altered pharmacokinetics of these agents with large convective volumes as well as hemoconcentration in the dialyzer in post-dilution mode

*HDF*, hemodiafiltration; *FF*, filtration fraction; *CVC*, central venous catheter; *AVF*, arteriovenous fistula

A blood flow of 5 to 8 mL/min/kg body weight or 150 to 250 mL/m^2^ body surface area per minute is required for HDF. When HDF treatment is initiated, a starting blood flow rate of 90 to 100 mL/min in the first HDF sessions is suggested, with increments of 10 mL/min/m^2^ body surface area (BSA) per week up to 200–250 mL/m^2^/min as tolerated.

### HDF vs. conventional HD—mechanisms of improved effects

The following key mechanisms are thought to account for the benefits of HDF over conventional HD:

I. *Clearance of toxins across a wide molecular weight range leading to improved dialysis efficiency*: Systemic inflammation, endothelial dysfunction, and oxidative stress that are seen in patients on dialysis may be due to circulating toxins such as β2M, retinol-binding protein, adiponectin, leptin, ghrelin, cholecystokinin, and cystatin C [27, 33]. HDF clears middle molecular weight toxins far more effectively than conventional HD; clearance of the prototype middle molecule β2M is 70–80% higher compared to HD [34]. Other middle molecules such as inflammatory cytokines that are involved in inflammation and oxidative stress are also cleared by HDF [35]. Furthermore, in the 3H study, it was shown that patients on HDF who lost residual kidney function did not show an increase in ß2M, whereas an increase was noted in children on HD [10].
II. *Improved hemodynamic stability*: HDF provides an innate cooling of the dialysate which may improve intradialytic hemodynamic stability [36], reduce rates of intradialytic hypotension [26], and result in fewer strokes [7] and a faster recovery time post-dialysis [10].
III. *Biocompatibility and reduced inflammation*: An increased removal of inflammatory cytokines by HDF contributes to reduced inflammation and oxidative stress [16].

Given that HDF treatment achieves superior clearances of several uremic toxins, some patients, particularly those who are late presenters to dialysis, may not tolerate HDF well. These patients may require short daily HD with slow blood flows and a gradual build-up to HDF treatment over a few weeks.

The 3H [10] and SWITCH [35] studies have documented reduced inflammation, oxidative stress and endothelial dysfunction, outcomes that are closely linked to the evolution and outcomes of cardiovascular disease, as well as growth, nutrition, and bone health, with HDF treatment compared to HD. Conventional HD is known to cause a pro-inflammatory milieu due to increased production and reduced clearance of inflammatory cytokines by diffusive therapy alone [11, 19]. HDF removes large middle-sized molecules as well as reduces the production of these molecules in the more biocompatible milieu [30]. Inflammatory cytokines such as high sensitivity CRP, TNF-α, and IL-6 were higher in HD compared to HDF patients even at baseline [10, 37, 38]. In the HD cohort, IL-6 and hs-CRP increased over the 12-month study period, while consistently lower levels were seen in the HDF cohort over the 12-month follow-up [10, 37, 38], which is consistent with results from trials in adults [32]. Accordingly, Agbas et al. showed that within just 3 months of switching HD patients to HDF with all other dialysis-related parameters left unchanged, a significant improvement in the endothelial risk profile was noted, perhaps due to a decrease in inflammation and increase in antioxidant capacity [35]. This is in line with other trials reporting significantly lower values of high sensitivity CRP, IL-6, TNF-α, and ß2M in the long term in pediatric patients who switched from HD to HDF [6, 11, 19].

## Clinical outcomes of HDF compared to HD in adults on dialysis

Observational studies, registries, and RCTs provide conflicting results on the outcomes of HDF, which to some extent can be explained by differences in the HD (low vs. high flux) and HDF techniques, variations in the type of vascular access, treatment time, actual delivered convective volume, and patient demographics [7]. A Cochrane review performed over a decade ago combined outcomes of both HF and HDF studies as “convective therapies” without differentiating the convective volumes achieved and did not show a benefit of HDF over HD [39], highlighting that not all convective therapies are equal.

In adults, the question of improved cardiovascular outcomes and a possible survival benefit in patients treated with OL-HDF compared to those treated with HD was addressed in five RCTs from Europe [7, 8, 26–28]. Only two RCTs, the Estudio de Supervivencia de Hemodiafiltración On-line (ESHOL) [7] and comparison of high-dose HDF with high-flux HD (CONVINCE) [8] trials showed an a priori benefit of HDF over HD. In the ESHOL trial, convective volumes of 23 L/session were achieved and a survival benefit of high-volume HDF compared to high-flux HD was shown [7]. Some of the early studies in HDF in adults including the (CONTRAST) Convective Transport study [27], On-line Hemodiafiltration study from Turkey [28], and the FRENCHIE study in elderly dialysis patients [26] aimed for and achieved lower convective volumes, and could not show an a priori benefit in improving all-cause or cardiovascular mortality. However, both the Turkish [28] and CONTRAST [27] studies on post hoc analysis showed that HDF patients who achieved a convective volume above > 17.4 L/session in the Turkish study [28] and > 20 L/session in the CONTRAST study [27] had lower all-cause and cardiovascular mortality. A pooled individual participant data analysis from four large RCTs [7, 26–28], confirmed a risk reduction of 14% for all-cause mortality and 23% for cardiovascular mortality by OL-HDF compared to conventional HD [29] with a dose-response relationship between the convective volume and survival.

The recently published CONVINCE trial has addressed survival outcomes in patients on OL-HDF who achieve optimal convective volume of ≥ 23 L/session; this was a key inclusion criterion to the study, and the mean achieved convective volume was 25.3 L per session [8]. With 1360 patients from 61 dialysis centers in eight European countries randomized to receive high-dose HDF or high-flux HD [40], over a median follow-up of 30 months, the risk of death was 23% higher in patients receiving high-flux HD compared to those receiving high-dose HDF. In pre-determined subgroup analyses, the mortality was significantly lower in those without pre-existing cardiovascular disease or diabetes, patients dialyzing through an arteriovenous fistula, over 65 year olds and those with a dialysis vintage of less than 2 years, suggesting that patients with fewer comorbidities and very good vascular access are likely to have better outcomes with HDF compared to high-flux HD [41], implying that data from this study cannot be generalized to the wider dialysis population.

## Outcomes in children

Several pediatric studies have compared HDF to conventional HD, including low- and high-flux HD modalities. HDF is now widely used across many centers in Europe, Canada and Asia, with 58% of children in western Europe on HDF (personal communication from the International Pediatric Hemodialysis Network [IPHN] Registry). As reported from the Italian Registry, HDF use may be limited to approximately 25% of patients on extracorporeal dialysis, in particular to those with high dialysis vintage and/ or those in whom a long waiting time to kidney transplantation is anticipated [42]. Data from the IPHN has shown that the global prevalence of HDF use is limited with only 15% of children around the world on HDF.

**Fig. 3:**
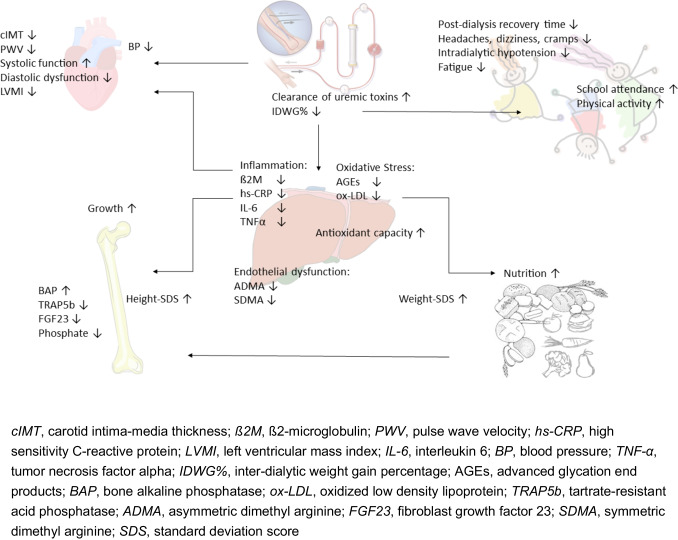
Outcomes of HDF in children

While many studies in pediatric HDF have been small, single-center, and cross-sectional, the 3H study is a multi-center, non-randomized parallel-arm intervention study that has prospectively studied nearly 40% of all children on extracorporeal dialysis across 10 countries in Europe and North America [10]. Both incident and prevalent patients between 5 and 20 years of age undergoing post-dilution HDF or HD on a 4-h per session 3 times per week schedule were included. The decision to perform HD or HDF was left to the treating physicians and based on usual center practice. Efforts to achieve the highest possible blood flow rate in both groups and a target convection volume of 12–15 L/m^2^ BSA in the HDF cohort were goals. The co-primary end points were an annualized change in carotid intima-media thickness (cIMT) standard deviation score (SDS) and height-SDS. Multiple exploratory end points relating to cardiovascular measures, nutrition and growth, and quality of life were assessed. Key findings from all pediatric studies are described below and summarized in Table 2 and Fig. 3.

**Table 2:** Key findings from pediatric studies

Title	Author, year of publication	Patients (n)	Study design	Objectives	Outcomes
Daily online hemodiafiltration promotes catch-up growth in children on chronic dialysis	Fischbach et al. 2010, [9]	15	Single-center observational prospective non-randomized study	Assessment of nutritional status and growth outcome	• Catch-up growth in first year of HDF • Significant increase in height SDS • Increase in mean BMI • Decrease of MAP
Role of online hemodiafiltration in improvement of inflammatory status in children with ESRD	Morad et al. 2014, [19]	30	Single-center cohort study HDF vs. conventional HD	Assessment of inflammatory risk associated with HDF compared to conventional HD	• Decrease of hs-CRP, IL-6, and TNF-α
The effect of on-line hemodiafiltration on improving the cardiovascular function parameters in children on regular dialysis	Fadel et al. 2015, [6]	30	Prospective comparatory study Pre-dilution HDF vs. conventional low-flux HD	Assessment of the effect of HDF on improving the chronic inflammatory state associated with CKD and the possible impact of these changes on myocardial function in children on chronic HD	• Decrease in hs-CRP • Decrease in frequency of diastolic dysfunction • Improvement of systolic function
Hemodiafiltration is associated with reduced inflammation, oxidative stress and improved endothelial risk profile compared to high-flux hemodialysis in children	Ağbaş et al. 2018, [35]	22	Prospective observational study Post-dilution HDF vs. high-flux HD	Determination of the intra-individual changes in markers of oxidative stress, total antioxidant capacity, inflammation, and endothelial dysfunction, on different dialysis modalities	• Significant reduction in ß2M, hs-CRP, ADMA, SDMA, AGEs, ox-LDL • Significant increase in TAC
Effects of hemodiafiltration versus conventional hemodialysis in children with ESKD: the HDF, Heart and Height Study	Shroff et al. 2019, [10]	177	Multi-centre, prospective, non-randomized parallel-arm intervention study Post-dilution HDF vs. high-flux HD	Testing the hypothesis that children on HDF have an improved cardiovascular risk profile, growth and nutritional status and quality of life, compared to those on conventional HD	Primary outcome measures: • Decrease in cIMT-SDS • Increase in height SDS Secondary outcome measures: • Lower IDWG% • Decrease in PWV SDS • Lower MAP-SDS • Lower LVMI • Lower ß2M • Lower hs-CRP • Decrease in PTH levels • Increase in hemoglobin • Shorter post-dialysis recovery time • Increased physical activity • Improved school attendance • Less headaches, dizziness, cramps
Online Hemodiafiltration use in children: a single center experience with a twist	Ibrahim et al. 2020, [11]	34	Sequential (2 phases) clinical follow-up study Randomized controlled Conventional HD vs. once-weekly and twice-weekly post-dilution HDF	To evaluate the benefits of incorporating HDF with different regimens in the treatment of children with CKD stage 5	• Higher weight SDS • Higher height SDS • Improvement of anemia • Lower hs-CRP, ß2M and IL-6 • Higher Kt/V/week • Decrease in percent change of post-dialysis fatigue frequency • Improvement on blood pressure control • Decrease in non-dialysis costs • Positive benefit score in all HDF patients
Hemodiafiltration maintains a sustained improvement in BP compared to conventional hemodialysis in children—the HDF, Heart and Height (3H) study	De Zan et al. 2020, [46]	78	Post hoc analysis of the 3H study	Determination of the risk factors associated with the evolution of BP, evaluation of BP control and effect of antihypertensive medications on BP control	• Lower 24-h MAP-SDS • MAP-SDS increased by 0.98 SDS in HD and non-significant by 0.15 SDS in HDF • IDWG% = risk factor for higher 24-h MAP • 88% on HD and 42% on HDF had MAP-SDS > 95th percentile at 12 months • Lower MAP-SDS in all age groups • Uncontrolled hypertension was present in 93% on HD and 38% on HDF
Hemodiafiltration is associated with reduced inflammation and increased bone formation compared with conventional hemodialysis in children: the HDF, Heart and Height (3H) study	Fischer et al. 2021, [38]	144	Post hoc analysis of the 3H study	Determination of the prevalence and risk factors for CKD-related bone disease and the changes in bone biomarkers in children on HDF and conventional HD and the effect of different dialysis modalities on the evolution of MBD	Patients on HDF compared to HD have: • No difference in biomarkers of CKD-MBD • Lower IL-6, TNF-α, hs-CRP • Higher Fetuin-A • Weak inverse correlation of serum phosphate levels with height SDS • Increase in BAP z-score • Lower TRAP5b z-scores • Lower sclerostin • Increase of BAP/TRAP5b ratio • Decrease in FGF23 by 25% • Lower FGF23/klotho ratio
Nutritional and Anthropometric Indices in Children receiving Haemodiafiltration vs Conventional Hemodialysis—the HDF, Heart and Height (3H) study	Paglialonga et al. 2023, [37]	107	Post hoc analysis of the 3H study	Examination of the correlation between auxological parameters and nutrition-related biomarkers in children on HD or HDF	Patients on HDF compared to HD have: • Higher residual renal output • Higher Ht SDS • Higher Wt SDS • Higher annualized change in Wt SDS • Higher serum albumin • Higher serum bicarbonate • Lower ß2M, hs-CRP and IL-6 values

### Cardiovascular outcomes

Children are uniquely suited to study the effects of dialysis treatment due to the high prevalence of sub-clinical cardiovascular disease [42] and the absence of other health issues such as diabetes or hypertension that are typically present in adults [43]. A change in cardiovascular outcomes (carotid intima-media thickness (cIMT), pulse wave velocity (PWV), and left ventricular mass index (LVMI)) on HDF compared to HD was a primary outcome measure of the 3H study [10]. Within 1 year of HD, the cIMT increased by 0.41 SDS, whereas there was no change in HDF patients [10]. Propensity score analysis showed that children on HD had a + 0.47 greater increase in annualized cIMT-SDS change (95% CI 0.07 to 0.87; *p* = 0.02) compared to those on HDF. Clearance of middle molecular weight uremic toxins as well as improved fluid removal by HDF were correlated with improved vascular outcomes in HDF [10]. Aortic stiffness, a consequence of arteriosclerosis and vascular calcification, correlated with the improved fluid control on HDF [10]. The LVMI was higher in HD compared to HDF patients at 12 months and correlated with the improved fluid control as well as higher hemoglobin and a lower PTH on HDF [10]. Similarly, Fadel et al. have shown that within a 6-month period of moving children from HD to HDF, systolic function improved and diastolic dysfunction decreased, but left ventricular mass was unchanged [6]. Inflammatory processes are also important contributors to cardiovascular morbidities and described by several authors as part of the “non-traditional risk factors” for cardiovascular disease [35, 44, 45]. An early and sustained attenuation of inflammatory markers is seen in patients on HDF compared to HD treatment [6, 35].

### Blood pressure control

In the 3H study, 24-h ambulatory blood pressure recordings were performed at baseline and 12-month follow-up. The mean arterial pressure (MAP)-SDS was higher and increased more rapidly in children on conventional HD compared to those on HDF [10]. Over a 1-year follow-up, there was a non-significant increase in the MAP of 0.15 SDS in children on HDF, whereas the MAP increased by 0.98 SDS in HD patients [46]. The improved BP control and lower inter-dialytic weight gain in patients on HDF are likely due to improved sodium mass transfer and tolerance of UF due to fewer episodes of intradialytic hypotension.

Furthermore, uncontrolled hypertension was far more common in children on HD compared to those on HDF, and no benefit was seen with anti-hypertension medications [46]. Small single-center pediatric studies have not reported significant differences between conventional HD and HDF [6, 11, 19]. An observational study suggests that BP, phosphate, and PTH control improved when children were moved from nocturnal in-center HD to nocturnal in-center HDF [47].

Importantly, HDF causes fewer intradialytic hemodynamic changes such as intradialytic hypotension than conventional HD and is therefore a safe and well-tolerated regimen [6]. A lower inter-dialytic weight gain in patients on HDF was directly associated with fewer intradialytic hypotensive episodes, shorter post-dialysis recovery time, and fewer post-dialysis symptoms such as headaches, dizziness, and cramps [46]. Improved intradialytic hemodynamic stability in HDF has also been noted in adult studies [7, 26], with a reduced risk of strokes.

### Bone health

Skeletal problems such as fractures and deformities are common in patients on dialysis [38, 48]. The 3H study investigated circulating biomarkers of bone turnover including bone formation marker bone-specific alkaline phosphatase (BAP) and bone resorption marker tartrate-resistant acid phosphatase 5b (TRAP5b). The ratio of the enzymatic activity of BAP/TRAP5b, implying net bone formation, increased in HDF patients to a level comparable to healthy children, but remained unchanged in HD over 12 months [38]. The fibroblast growth factor 23 (FGF23), a middle molecular weight toxin, showed a 25% reduction in patients on HDF, whereas levels increased by over 100% in children on HD [38]. Although the impact of FGF23 on bone health in children is yet to be determined, FGF23 is known to have several “off-target” effects on cardiac myocytes [49] with an increased prevalence of left ventricular hypertrophy. HDF achieves excellent convective clearance of FGF23, which in turn may partially explain the lower left ventricular mass in the 3H study [10] and improved cardiovascular outcomes in adults on HDF [7]. Some studies have also shown a reduction in serum phosphate and PTH levels with HDF vs. HD [11].

### Growth and nutritional parameters

The first reports on improved growth on HDF were from Fischbach et al. who showed a dramatic increase in the mean growth velocity during the first year of HDF [9]. However, these studies delivered a very high dialysis dose using six times per week HDF in pre-dilution mode, and most children also received growth hormone treatment, making it difficult to discern the benefits of HDF therapy alone. In the 3H study, patients on HDF experienced a small but statistically significant increase in the annualized change in height SDS while it remained static in patients on HD, independent of growth hormone treatment [10]. The increase in height SDS correlated with serum β2M concentrations, suggesting that clearance of middle MW compounds such as endogenous gonadotropin and somatomedin inhibitors as well as inflammatory cytokines may partly alleviate resistance to GH in patients on HDF [10], with HDF suggested to be the perfect “stimulus package” for growth [50]. These potential anabolic effects of HDF were further confirmed by Ibrahim et al. who showed that children on HDF had significantly higher height SDS and higher percent changes of height SDS and weight SDS compared to the HD group [11].

A further post hoc analysis of the 3H study has shown that a higher annualized increase in weight SDS was noted in HDF patients only. Des-acyl ghrelin was independently and negatively associated with height SDS and weight SDS but the study failed to demonstrate a better clearance of anorexigenic hormones by HDF compared to HD implying there might be other mechanisms responsible for this [37].

### Safety and tolerability

There were no differences in the rate of change of residual kidney function nor any reduction in serum albumin levels on HD or HDF treatments in the 3H trial [10].

Lower inter-dialytic weight gain on HDF was noted in the 3H trial. This implies lower ultrafiltration rates per session, in turn allowing for greater hemodynamic stability, and fewer adverse symptoms on dialysis [10]. Two RCTs in adults have shown similar benefits: improved intradialytic hemodynamic stability in HDF is likely to have led to fewer symptomatic intradialytic hypotensive episodes in the FRENCHIE study in a vulnerable population of elderly dialysis patients [26], and in the ESHOL study [7], although mechanisms for this are poorly understood [51]. However, when patients were blinded to dialysis type in a randomized cross-over trial, there was no difference in the patient-reported quality of life scores nor the post-dialysis recovery time [52].

### Health-related quality of life

Pursuing the goal of having researchers focus on valuable outcomes that are of importance to the patients, their families, and practitioners, the Standardized Outcomes in Nephrology (SONG-Kids) workgroup has created a list of outcomes, in which life participation is one of the four core outcomes [53]. HDF promoted “life participation” by improving school attendance and physical activity [10, 46]. Children in the 3H trial who were treated with HDF rather than conventional HD showed a reduction in the post-dialysis recovery time and had fewer incidences of headaches, dizziness, and cramps [10]. It is likely that lower ultrafiltration rates and better hemodynamic stability on HDF led to an improved vascular refilling during the dialysis session, which in turn reduced the propensity for hypotensive episodes [10].

Chronic fatigue, reported in up to 60–97% of patients on long-term dialysis [54], is one of the most common and distressing symptoms that limits the quality of life of patients and has been defined as a highly prioritized outcome in the SONG-Kids initiative [53]. A significant reduction in the percent change of post-dialysis fatigue frequency was shown both in the short and long term for HDF patients [11]. Reduced symptom burden with simultaneous increase in physical performance is evidence of good tolerability of treatment with HDF in children.

## Conclusion

The existing literature suggests significant potential benefits of HDF over HD in pediatric populations, although confirmation through randomized trials is required. The favorable biocompatible milieu, greater clearance of middle molecular weight uremic toxins, reduced inflammation, and hemodynamic stability contribute to lower levels of sub-clinical cardiovascular damage, improved blood pressure control, improved growth and bone health, and a better health-related quality of life. While clinical outcomes are of paramount importance, future studies should also integrate patient-centered outcomes, economic evaluations, and the environmental impact of different dialysis modalities.

## Key summary points

1. Children with stage 5 CKD on dialysis face high mortality and morbidity.
2. HDF, a combination of diffusive and convective transport, enhances clearance of middle molecular weight uremic toxins, including inflammatory cytokines, and provides intra-dialytic hemodynamic stability.
3. There is a dose-response relationship between convective volume and survival in adults on HDF.
4. Pediatric studies demonstrate attenuated cardiovascular and inflammatory risk profiles, improved growth, BP control, bone health, and an improved quality of life with HDF compared to HD therapy.
5. Patients receiving a short duration of dialysis and those with residual kidney function also have improved outcomes on HDF compared to HD.

## Multiple choice questions

Answers appear following the reference list.

1. Which mode of application of the replacement fluid in HDF is most commonly used in children and adults?
  - Pre-dilution
  - Post-dilution
  - Mixed-dilution
  - Mid-dilution
2. Which one of these is not a technical requirement specific for HDF in children?
  - Ultrapure water
  - High-flux dialyzer
  - Dialysis machines with accurate pressure control
  - Dialysis machines with accurate ultrafiltration control
3. Which one of these effects is not seen with HDF?
  - Reduction in residual kidney function
  - Clears some inflammatory cytokines
  - Reduces levels of FGF23
  - Reduces left ventricular mass index
4. Which of these substances is not cleared by HDF?
  - Interleukin-6
  - Fibroblast growth factor 23
  - Oxalate
  - Indoxyl sulphate
5. Which one of the following statements is true?
  - Post-dialysis recovery time is longer in HDF than in HD.
  - HDF has a catabolic effect.
  - In children a target convective volume of 13-15 L/m^2^/session is aimed for in post-dilution mode.
  - Uncontrolled hypertension is more common in children on HDF compared to HD.

## Abbreviations

HDF: Hemodiafiltration
HD: Hemodialysis
SDS: Standard deviation score
BMI: Body mass index
MAP: Mean arterial pressure
ESKD: End stage kidney disease
hs-CRP: High-sensitivity C-reactive protein
IL-6: Interleukin 6
TNF-α: Tumor necrosis factor alpha
β2M: Beta-2 microglobulin
ADMA: Asymmetric dimethyl arginine
SDMA: Symmetric dimethyl arginine
AGEs: Advanced glycation end-products
ox-LDL: Oxidized low density lipoprotein
cIMT: Carotid intima-media thickness
PWV: Pulse wave velocity
LVMI: Left ventricular mass index
PTH: Parathyroid hormone
IDWG%: Interdialytic weight gain percentage
BAP: Bone alkaline phosphatase
TRAP5b: Tartrate-resistant acid phosphatase
FGF23: Firoblast growth factor 23

## References

[1] Shroff R, Weaver DJ Jr, Mitsnefes MM (2011) Cardiovascular complications in children with chronic kidney disease. Nat Rev Nephrol 7:642–649 21912426 10.1038/nrneph.2011.116

[2] Tjaden LA, Grootenhuis MA, Noordzij M, Groothoff JW (2016) Health-related quality of life in patients with pediatric onset of end-stage renal disease: state of the art and recommendations for clinical practice. Pediatr Nephrol 31:1579–1591 26310616 10.1007/s00467-015-3186-3 PMC4995226

[3] Shroff RC, Donald AE, Hiorns MP, Watson A, Feather S, Milford D, Ellins EA, Storry C, Ridout D, Deanfield J, Rees L (2007) Mineral metabolism and vascular damage in children on dialysis. J Am Soc Nephrol 18:2996–3003 17942964 10.1681/ASN.2006121397

[4] Shroff RC, McNair R, Figg N, Skepper JN, Schurgers L, Gupta A, Hiorns M, Donald AE, Deanfield J, Rees L, Shanahan CM (2008) Dialysis accelerates medial vascular calcification in part by triggering smooth muscle cell apoptosis. Circulation 118:1748–1757 18838561 10.1161/CIRCULATIONAHA.108.783738

[5] Shroff R, Hothi D, Symons J (2022) Chronic hemodialysis in children. In: Emma F, Goldstein SL, Bagga A, Bates CM, Shroff R (eds) Pediatric nephrology. Springer International Publishing, Cham, pp 1835–1868

[6] Fadel FI, Makar SH, Zekri H, Ahmed DH, Aon AH (2015) The effect of on-line hemodiafiltration on improving the cardiovascular function parameters in children on regular dialysis. Saudi J Kidney Dis Transpl 26:39–46 25579714 10.4103/1319-2442.148731

[7] Maduell F, Moreso F, Pons M, Ramos R, Mora-Macià J, Carreras J, Soler J, Torres F, Campistol JM, Martinez-Castelao A (2013) High-efficiency postdilution online hemodiafiltration reduces all-cause mortality in hemodialysis patients. J Am Soc Nephrol 24:487–497 23411788 10.1681/ASN.2012080875 PMC3582206

[8] Blankestijn PJ, Vernooij RWM, Hockham C, Strippoli GFM, Canaud B, Hegbrant J, Barth C, Covic A, Cromm K, Cucui A, Davenport A, Rose M, Török M, Woodward M, Bots ML (2023) Effect of hemodiafiltration or hemodialysis on mortality in kidney failure. N Engl J Med 389:700–709 37326323 10.1056/NEJMoa2304820

[9] Fischbach M, Terzic J, Menouer S, Dheu C, Seuge L, Zalosczic A (2010) Daily on line haemodiafiltration promotes catch-up growth in children on chronic dialysis. Nephrol Dial Transplant 25:867–873 19889872 10.1093/ndt/gfp565

[10] Shroff R, Smith C, Ranchin B, Bayazit AK, Stefanidis CJ, Askiti V, Azukaitis K, Canpolat N, Agbas A, Aitkenhead H, Anarat A, Aoun B, Aofolaju D, Bakkaloglu SA, Bhowruth D, Borzych-Duzalka D, Bulut IK, Buscher R, Deanfield J, Dempster C, Duzova A, Habbig S, Hayes W, Hegde S, Krid S, Licht C, Litwin M, Mayes M, Mir S, Nemec R, Obrycki L, Paglialonga F, Picca S, Samaille C, Shenoy M, Sinha MD, Spasojevic B, Stronach L, Vidal E, Vondrak K, Yilmaz A, Zaloszyc A, Fischbach M, Schmitt CP, Schaefer F (2019) Effects of hemodiafiltration versus conventional hemodialysis in children with ESKD: the HDF, heart and height study. J Am Soc Nephrol 30:678–691 30846560 10.1681/ASN.2018100990 PMC6442347

[11] Ibrahim MAA, ElHakim IZ, Soliman D, Mubarak MA, Said RM (2020) Online hemodiafiltration use in children: a single center experience with a twist. BMC Nephrol 21:306 32723294 10.1186/s12882-020-01957-9 PMC7388526

[12] Ashby D, Borman N, Burton J, Corbett R, Davenport A, Farrington K, Flowers K, Fotheringham J, Andrea Fox RN, Franklin G, Gardiner C, Martin Gerrish RN, Greenwood S, Hothi D, Khares A, Koufaki P, Levy J, Lindley E, Macdonald J, Mafrici B, Mooney A, Tattersall J, Tyerman K, Villar E, Wilkie M (2019) Renal association clinical practice guideline on haemodialysis. BMC Nephrol 20:379 31623578 10.1186/s12882-019-1527-3 PMC6798406

[13] Tattersall J, Blankestijn, PJ (2023) Technical aspects of hemodiafitration. UpToDate. https://www.uptodate.com/contents/technical-aspects-of-hemodiafiltration. Accessed 4 May 2023

[14] Mostovaya IM, Blankestijn PJ, Bots ML, Covic A, Davenport A, Grooteman MP, Hegbrant J, Locatelli F, Vanholder R, Nubé MJ (2014) Clinical evidence on hemodiafiltration: a systematic review and a meta-analysis. Semin Dial 27:119–127 24738146 10.1111/sdi.12200

[15] Reis T, Ronco C, Soranno DE, Clark W, De Rosa S, Forni LG, Lorenzin A, Ricci Z, Villa G, Kellum JA, Mehta R, Rosner MH, Faculty Nomenclature Standardization (2023) Standardization of Nomenclature for the Mechanisms and Materials Utilized for Extracorporeal Blood Purification. Blood Purif. 10.1159/000533330 37703868 10.1159/000533330

[16] Ledebo I, Blankestijn PJ (2010) Haemodiafiltration-optimal efficiency and safety. NDT Plus 3:8–16 20090878 10.1093/ndtplus/sfp149 PMC2808132

[17] Tattersall JE, Ward RA, EUDIAL group, (2013) Online haemodiafiltration: definition, dose quantification and safety revisited. Nephrol Dial Transplant 28:542–550 23345621 10.1093/ndt/gfs530

[18] Snauwaert E, Van Biesen W, Raes A, Glorieux G, Vande Walle J, Roels S, Vanholder R, Askiti V, Azukaitis K, Bayazit A, Canpolat N, Fischbach M, Saoussen K, Litwin M, Obrycki L, Paglialonga F, Ranchin B, Samaille C, Schaefer F, Schmitt CP, Spasojevic B, Stefanidis CJ, Shroff R, Eloot S (2019) Haemodiafiltration does not lower protein-bound uraemic toxin levels compared with haemodialysis in a paediatric population. Nephrol Dial Transplant 35:648–656 10.1093/ndt/gfz132 31361315

[19] Morad AA, Bazaraa HM, Abdel Aziz RE, Abdel Halim DA, Shoman MG, Saleh ME (2014) Role of online hemodiafiltration in improvement of inflammatory status in pediatric patients with end-stage renal disease. Iran J Kidney Dis 8:481–485 25362224

[20] de Zan F, Schmitt CP, Shroff R (2022) Hemodiafiltration in the pediatric population. Semin Dial 35:427–430 36121112 10.1111/sdi.13072

[21] Ranchin B, Schmitt CP, Warady B, Craig JC, Licht C, Hataya H, Vidal E, Walle JV, Shroff R (2023) Devices for long-term hemodialysis in small children-a plea for action. Kidney Int 103:1038–1040 36990213 10.1016/j.kint.2023.03.018

[22] Marcelli D, Kopperschmidt P, Bayh I, Jirka T, Merello JI, Ponce P, Ladanyi E, Di Benedetto A, Dovc-Dimec R, Rosenberger J, Stuard S, Scholz C, Grassmann A, Canaud B (2015) Modifiable factors associated with achievement of high-volume post-dilution hemodiafiltration: results from an international study. Int J Artif Organs 38:244–250 26080930 10.5301/ijao.5000414

[23] Van Laecke S, De Wilde K, Vanholder R (2006) Online hemodiafiltration. Artif Organs 30:579–585 16911311 10.1111/j.1525-1594.2006.00266.x

[24] European Directorate for the Quality of Medicines (n.d.) Purified water. European Pharmacopoeia 9.4. https://www.edqm.eu/sites/default/files/institutional-brochure-edqm.pdf. Accessed 3 Sept 2023

[25] Canaud B, Davenport A (2022) Prescription of online hemodiafiltration (ol-HDF). Semin Dial 35:413–419 35297521 10.1111/sdi.13070

[26] Morena M, Jaussent A, Chalabi L, Leray-Moragues H, Chenine L, Debure A, Thibaudin D, Azzouz L, Patrier L, Maurice F, Nicoud P, Durand C, Seigneuric B, Dupuy AM, Picot MC, Cristol JP, Canaud B, FRENCHIE Study Investigators (2017) Treatment tolerance and patient-reported outcomes favor online hemodiafiltration compared to high-flux hemodialysis in the elderly. Kidney Int 91:1495–1509 28318624 10.1016/j.kint.2017.01.013

[27] Grooteman MP, van den Dorpel MA, Bots ML, Penne EL, van der Weerd NC, Mazairac AH, den Hoedt CH, van der Tweel I, Lévesque R, Nubé MJ, ter Wee PM, Blankestijn PJ (2012) Effect of online hemodiafiltration on all-cause mortality and cardiovascular outcomes. J Am Soc Nephrol 23:1087–1096 22539829 10.1681/ASN.2011121140 PMC3358764

[28] Ok E, Asci G, Toz H, Ok ES, Kircelli F, Yilmaz M, Hur E, Demirci MS, Demirci C, Duman S, Basci A, Adam SM, Isik IO, Zengin M, Suleymanlar G, Yilmaz ME, Ozkahya M (2013) Mortality and cardiovascular events in online haemodiafiltration (OL-HDF) compared with high-flux dialysis: results from the Turkish OL-HDF Study. Nephrol Dial Transplant 28:192–202 23229932 10.1093/ndt/gfs407

[29] Peters SA, Bots ML, Canaud B, Davenport A, Grooteman MP, Kircelli F, Locatelli F, Maduell F, Morena M, Nube MJ, Ok E, Torres F, Woodward M, Blankestijn PJ, HDF Pooling Project Investigators (2016) Haemodiafiltration and mortality in end-stage kidney disease patients: a pooled individual participant data analysis from four randomized controlled trials. Nephrol Dial Transplant 31:978–984 26492924 10.1093/ndt/gfv349

[30] Shroff R, Bayazit A, Stefanidis CJ, Askiti V, Azukaitis K, Canpolat N, Agbas A, Anarat A, Aoun B, Bakkaloglu S, Bhowruth D, Borzych-Duzalka D, Bulut IK, Buscher R, Dempster C, Duzova A, Habbig S, Hayes W, Hegde S, Krid S, Licht C, Litwin M, Mayes M, Mir S, Nemec R, Obrycki L, Paglialonga F, Picca S, Ranchin B, Samaille C, Shenoy M, Sinha M, Smith C, Spasojevic B, Vidal E, Vondrak K, Yilmaz A, Zaloszyc A, Fischbach M, Schaefer F, Schmitt CP (2018) Effect of haemodiafiltration vs conventional haemodialysis on growth and cardiovascular outcomes in children - the HDF, heart and height (3H) study. BMC Nephrol 19:199. 10.1186/s12882-018-0998-y 30097064 10.1186/s12882-018-0998-y PMC6086045

[31] Kikuchi K, Hamano T, Wada A, Nakai S, Masakane I (2019) Predilution online hemodiafiltration is associated with improved survival compared with hemodialysis. Kidney Int 95:929–938 30782421 10.1016/j.kint.2018.10.036

[32] den Hoedt CH, Bots ML, Grooteman MP, van der Weerd NC, Mazairac AH, Penne EL, Levesque R, ter Wee PM, Nubé MJ, Blankestijn PJ, van den Dorpel MA (2014) Online hemodiafiltration reduces systemic inflammation compared to low-flux hemodialysis. Kidney Int 86:423–432 24552852 10.1038/ki.2014.9

[33] Vernooij RWM, Hockham C, Barth C, Canaud B, Cromm K, Davenport A, Hegbrant J, Rose M, Strippoli GFM, Török M, Woodward M, Bots ML, Blankestijn PJ (2023) High-target hemodiafiltration convective dose achieved in most patients in a 6-month intermediary analysis of the CONVINCE randomized controlled trial. Kidney Int Rep 8:2276–2283 38025213 10.1016/j.ekir.2023.08.004 PMC10658200

[34] Cheung AK, Rocco MV, Yan G, Leypoldt JK, Levin NW, Greene T, Agodoa L, Bailey J, Beck GJ, Clark W, Levey AS, Ornt DB, Schulman G, Schwab S, Teehan B, Eknoyan G (2006) Serum beta-2 microglobulin levels predict mortality in dialysis patients: results of the HEMO study. J Am Soc Nephrol 17:546–555 16382021 10.1681/ASN.2005020132

[35] Ağbaş A, Canpolat N, Çalışkan S, Yılmaz A, Ekmekçi H, Mayes M, Aitkenhead H, Schaefer F, Sever L, Shroff R (2018) Hemodiafiltration is associated with reduced inflammation, oxidative stress and improved endothelial risk profile compared to high-flux hemodialysis in children. PLoS One 13:e0198320 29912924 10.1371/journal.pone.0198320 PMC6005477

[36] Locatelli F, Altieri P, Andrulli S, Bolasco P, Sau G, Pedrini LA, Basile C, David S, Feriani M, Montagna G, Di Iorio BR, Memoli B, Cravero R, Battaglia G, Zoccali C (2010) Hemofiltration and hemodiafiltration reduce intradialytic hypotension in ESRD. J Am Soc Nephrol 21:1798–1807 20813866 10.1681/ASN.2010030280 PMC3013537

[37] Paglialonga F, Monzani A, Prodam F, Smith C, De Zan F, Canpolat N, Agbas A, Bayazit A, Anarat A, Bakkaloglu SA, Askiti V, Stefanidis CJ, Azukaitis K, Bulut IK, Borzych-Dużałka D, Duzova A, Habbig S, Krid S, Licht C, Litwin M, Obrycki L, Ranchin B, Samaille C, Shenoy M, Sinha MD, Spasojevic B, Vidal E, Yilmaz A, Fischbach M, Schaefer F, Schmitt CP, Edefonti A, Shroff R (2023) Nutritional and anthropometric indices in children receiving haemodiafiltration vs conventional haemodialysis - the HDF, Heart and Height (3H) Study. J Ren Nutr 33:17–28 35870690 10.1053/j.jrn.2022.07.005

[38] Fischer DC, Smith C, De Zan F, Bacchetta J, Bakkaloglu SA, Agbas A, Anarat A, Aoun B, Askiti V, Azukaitis K, Bayazit A, Bulut IK, Canpolat N, Borzych-Duzalka D, Duzova A, Habbig S, Krid S, Licht C, Litwin M, Obrycki L, Paglialonga F, Rahn A, Ranchin B, Samaille C, Shenoy M, Sinha MD, Spasojevic B, Stefanidis CJ, Vidal E, Yilmaz A, Fischbach M, Schaefer F, Schmitt CP, Shroff R (2021) Hemodiafiltration is associated with reduced inflammation and increased bone formation compared with conventional hemodialysis in children: the HDF, hearts and heights (3H) study. Kidney Int Rep 6:2358–2370 34514197 10.1016/j.ekir.2021.06.025 PMC8418977

[39] Nistor I, Palmer SC, Craig JC, Saglimbene V, Vecchio M, Covic A, Strippoli GF (2015) Haemodiafiltration, haemofiltration and haemodialysis for end-stage kidney disease. Cochrane Database Syst Rev: CD006258 10.1002/14651858.CD006258.pub2 PMC10766139 25993563

[40] Blankestijn PJ, Fischer KI, Barth C, Cromm K, Canaud B, Davenport A, Grobbee DE, Hegbrant J, Roes KC, Rose M, Strippoli GF, Vernooij RW, Woodward M, de Wit GAd, Bots ML (2020) Benefits and harms of high-dose haemodiafiltration versus high-flux haemodialysis: the comparison of high-dose haemodiafiltration with high-flux haemodialysis (CONVINCE) trial protocol. BMJ Open 10:e033228 32029487 10.1136/bmjopen-2019-033228 PMC7044930

[41] Shroff R, Basile C, van der Sande F, Mitra S (2023) Haemodiafiltration for all: are we CONVINCEd? Nephrol Dial Transplant 38:2663–2665 37391380 10.1093/ndt/gfad136

[42] Paglialonga F, Vidal E, Pecoraro C, Guzzo I, Giordano M, Gianoglio B, Corrado C, Roperto R, Ratsch I, Luzio S, Murer L, Consolo S, Pieri G, Montini G, Edefonti A, Verrina E (2019) Haemodiafiltration use in children: data from the Italian Pediatric Dialysis Registry. Pediatr Nephrol 34:1057–1063 30612203 10.1007/s00467-018-4184-z

[43] Querfeld U, Anarat A, Bayazit AK, Bakkaloglu AS, Bilginer Y, Caliskan S, Civilibal M, Doyon A, Duzova A, Kracht D, Litwin M, Melk A, Mir S, Sozeri B, Shroff R, Zeller R, Wuhl E, Schaefer F (2010) The Cardiovascular comorbidity in children with chronic kidney disease (4C) study: objectives, design, and methodology. Clin J Am Soc Nephrol 5:1642–1648 20576824 10.2215/CJN.08791209 PMC2974406

[44] Civilibal M, Caliskan S, Oflaz H, Sever L, Candan C, Canpolat N, Kasapcopur O, Bugra Z, Arisoy N (2007) Traditional and “new” cardiovascular risk markers and factors in pediatric dialysis patients. Pediatr Nephrol 22:1021–1029 17340147 10.1007/s00467-007-0451-0

[45] Dai L, Golembiewska E, Lindholm B, Stenvinkel P (2017) End-stage renal disease, inflammation and cardiovascular outcomes. Contrib Nephrol 191:32–43 28910789 10.1159/000479254

[46] De Zan F, Smith C, Duzova A, Bayazit A, Stefanidis CJ, Askiti V, Azukaitis K, Canpolat N, Agbas A, Anarat A, Aoun B, Bakkaloglu SA, Borzych-Duzalka D, Bulut IK, Habbig S, Krid S, Licht C, Litwin M, Obrycki L, Paglialonga F, Ranchin B, Samaille C, Shenoy M, Sinha MD, Spasojevic B, Yilmaz A, Fischbach M, Schmitt CP, Schaefer F, Vidal E, Shroff R (2021) Hemodiafiltration maintains a sustained improvement in blood pressure compared to conventional hemodialysis in children-the HDF, heart and height (3H) study. Pediatr Nephrol 36:2393–2403 33629141 10.1007/s00467-021-04930-2

[47] Thumfart J, Puttkamer CV, Wagner S, Querfeld U, Muller D (2014) Hemodiafiltration in a pediatric nocturnal dialysis program. Pediatr Nephrol 29:1411–1416 24535110 10.1007/s00467-014-2776-9

[48] Lalayiannis AD, Soeiro EMD, Moyses RMA, Shroff R (2023) Chronic kidney disease mineral bone disorder in childhood and young adulthood: a ‘growing’ understanding. Pediatr Nephrol. 10.1007/s00467-023-06109-3 37624528 10.1007/s00467-023-06109-3 PMC10817832

[49] Faul C, Amaral AP, Oskouei B, Hu MC, Sloan A, Isakova T, Gutierrez OM, Aguillon-Prada R, Lincoln J, Hare JM, Mundel P, Morales A, Scialla J, Fischer M, Soliman EZ, Chen J, Go AS, Rosas SE, Nessel L, Townsend RR, Feldman HI, St John Sutton M, Ojo A, Gadegbeku C, Di Marco GS, Reuter S, Kentrup D, Tiemann K, Brand M, Hill JA, Moe OW, Kuro OM, Kusek JW, Keane MG, Wolf M (2011) FGF23 induces left ventricular hypertrophy. J Clin Invest 121:4393–4408 21985788 10.1172/JCI46122 PMC3204831

[50] Schaefer F (2010) Daily online haemodiafiltration: the perfect ‘stimulus package’ to induce growth? Nephrol Dial Transplant 25:658–660 20083477 10.1093/ndt/gfp769

[51] Farrington K, Davenport A (2013) The ESHOL study: hemodiafiltration improves survival-but how? Kidney Int 83:979–981 23575565 10.1038/ki.2013.109

[52] Smith JR, Zimmer N, Bell E, Francq BG, McConnachie A, Mactier R (2017) A randomized, single-blind, crossover trial of recovery time in high-flux hemodialysis and hemodiafiltration. Am J Kidney Dis 69:762–770 28024931 10.1053/j.ajkd.2016.10.025 PMC5438239

[53] Hanson CS, Craig JC, Logeman C, Sinha A, Dart A, Eddy AA, Guha C, Gipson DS, Bockenhauer D, Yap HK, Groothoff J, Zappitelli M, Webb NJA, Alexander SI, Furth SL, Samuel S, Neu A, Viecelli AK, Ju A, Sharma A, Au EH, Desmond H, Shen JI, Manera KE, Azukaitis K, Dunn L, Carter SA, Gutman T, Cho Y, Walker A, Francis A, Sanchez-Kazi C, Kausman J, Pearl M, Benador N, Sahney S, Tong A, SONG-Kids consensus workshops investigators (2020) Establishing core outcome domains in pediatric kidney disease: report of the Standardized Outcomes in Nephrology-Children and Adolescents (SONG-KIDS) consensus workshops. Kidney Int 98:553–565 32628942 10.1016/j.kint.2020.05.054

[54] Ramadan Y, Elkoofy N, Sabry S, Mansour G, El-Anwar N (2023) Fatigue assessment and its predictors in pediatric patients with chronic kidney disease stages III to V. Egypt Paediatr Assoc Gazette 71:3–7 10.1186/s43054-022-00155-6

